# IRE1 Alpha/XBP1 Axis Sustains Primary Effusion Lymphoma Cell Survival by Promoting Cytokine Release and STAT3 Activation

**DOI:** 10.3390/biomedicines9020118

**Published:** 2021-01-27

**Authors:** Roberta Gonnella, Maria Saveria Gilardini Montani, Luisa Guttieri, Maria Anele Romeo, Roberta Santarelli, Mara Cirone

**Affiliations:** 1 Department of Experimental Medicine, Sapienza University of Rome, 00161 Rome, Italy; roberta.gonnella@uniroma1.it (R.G.); mariasaveria.gilardinimontani@uniroma1.it (M.S.G.M.); mariaanele.romeo@uniroma1.it (M.A.R.); roberta.santarelli@uniroma1.it (R.S.); 2 Laboratory Affiliated to Istituto Pasteur Italia-Fondazione Cenci Bolognetti, 00161 Rome, Italy; luisa.guttieri@uniroma1.it; 3 Department of Clinical and Molecular Medicine, Sapienza University of Rome, 00161 Rome, Italy

**Keywords:** ER stress, UPR, IRE1 alpha, XBP1, STAT3, cytokines

## Abstract

Primary Effusion Lymphoma (PEL) is a highly aggressive B cell lymphoma associated with Kaposi’s Sarcoma-associated Herpesvirus (KSHV). It is characterized by a high level of basal Endoplasmic Reticulum (ER) stress, Unfolded Protein Response (UPR) activation and constitutive phosphorylation of oncogenic pathways such as the Signal Transducer and activator of Transcription (STAT3). In this study, we found that the inositol requiring kinase (IRE) 1alpha/X-box binding protein (XBP1) axis of UPR plays a key role in the survival of PEL cells, while double stranded RNA-activated protein kinase-like ER kinase (PERK) and activating transcription factor (ATF) 6 slightly influence it, in correlation with the capacity of the IRE1alpha/XBP1 axis to induce the release of interleukin (IL)-6, IL-10 and Vascular-Endothelial Growth Factor (VEGF). Moreover, we found that IRE1alpha/XBP1 inhibition reduced STAT3 Tyr705 phosphorylation and induced a pro-survival autophagy in PEL cells. In conclusion, this study suggests that targeting the IRE1alpha/XBP1 axis represents a promising strategy against PEL cells and that the cytotoxic effect of this treatment may be potentiated by autophagy inhibition.

## 1. Introduction

Primary effusion lymphoma (PEL) is a highly aggressive B cell lymphoma whose etiology is strongly linked to Kaposi’s Sarcoma-associated herpesvirus (KSHV) infection that is present in almost 100% of cases of PEL, in the majority of cases in association with Epstein-Barr virus (EBV). KSHV is a gammaherpesvirus that carries genes encoding for a variety of proteins involved in tumorigenesis such as viral Fas-associated with death domain (FADD)-like interleukin-1β-converting enzyme (FLICE)/caspase 8–inhibitory protein (vFLIP) that is able to subvert the response to apoptotic stimuli and autophagy [1,2]. PEL presents in most cases as malignant lymphomatous effusions, mainly localized in body cavities, such as the pleural, pericardium and peritoneal cavities. PEL cells originate from postgerminal center late-differentiating B-cells [3]. The success of conventional therapies such as cyclophosphamide, doxorubicin, vincristine and prednisone (CHOP) against this lymphoma is very poor, which implies that the search for new and more effective treatments is urgent.

Autophagy, a catabolic process required for cellular homeostasis and response to stress, is important for cancer cell survival, especially in stressful conditions such as nutrient shortage or anticancer treatments. Autophagy inhibition in PEL cells in the course of several chemotherapies has been shown by our and others’ laboratories to be a promising strategy to improve the outcome of such treatments [4,5]. Moreover, similarly to multiple myeloma (MM), PEL cells are highly secretory B cells and, therefore, are characterized by basal Endoplasmic Reticulum (ER) stress and Unfolded Protein Response (UPR) activation [6], which can be also increased by treatments, particularly the proteasome inhibitor Bortezomib [7]. Although a too high stress and too prolonged UPR activation may induce cell death, UPR is mainly a response that can help cells to survive by several means, i.e., by increasing chaperone expression, by blocking protein translation and degradation by proteasome and autophagy [8]. All these processes may be triggered by the single or overlapping functions of the three main UPR sensors, namely, inositol requiring kinase (IRE) 1 alpha, double stranded RNA-activated protein kinase-like ER kinase (PERK) and the activating transcription factor (ATF) 6, that indeed orchestrate an integrate response to stress. Regarding Ire1 alpha, this sensor may have kinase and endoribonuclease activities, generating in the latter case the spliced form of XBP1 (XBP1s), a molecule strongly involved in the survival of cancers such as MM [9]. Of note, UPR signaling affects multiple other processes, playing a key role in cellular and organism pathophysiology. UPR regulates, for example, the activation of molecular pathways such as NF-kB and STAT3 [10] that, among other functions, promote the release of cytokines and affect cancer cell survival as well as immune response [11]. Moreover, the antioxidant response, essential for maintaining the proper ROS balance required for the activation of pro-survival intracellular signaling, is also strongly influenced by UPR activation, particularly by PERK that may activate nuclear factor erythroid 2-related factor (NRF2) [12]. Given the central role of UPR in regulating multiple processes that ultimately dictate the cell fate, in this study, we investigated the possibility to reduce survival of PEL cells, characterized by a basal ER stress/UPR activation by inhibiting the tree UPR sensors PERK, IRE1alpha or ATF6. We also explored the impact of UPR sensor inhibition on autophagy, knowing that all these sensors have been shown to be involved in the activation of this catabolic process [13].

## 2. Material and Methods

### 2.1. Cell Culture and Reagents

BC3 (ATCC, CRL-2277) and BCBL1 (kindly provided by Prof. P. Monini, National AIDS Center, Istituto Superiore di Sanità, Rome, Italy) are human B-cell lines derived from Primary effusion Lymphoma (PEL). Cells were cultured in RPMI 1640 (Sigma-Aldrich, St. Louis, MO, USA, R0883) with 10% fetal calf serum (Euroclone, Milano, Italy, ECLS0180L), l-glutamine (2 mM) streptomycin (100 µg/mL) and penicillin (100 U/mL) (Gibco, Gaithersburg, MD, USA, 10378-016) in 5% CO_2_ at 37 °C. Cells were treated with the following drugs: 4μ8C (IRE1 RNAse inhibitor) provided by Sigma (Sigma-Aldrich, MO, USA, cat n. SML0949), Ceapin-A7 (ATF6a signaling blocker) provided by Sigma-Aldrich (cat n. SML2330), GSK2606414 (PERK inhibitor) provided by Selleckem, USA (cat. n. S7307). Chloroquine (CQ) (inhibitor of autophagic protein degradation) (Sigma-Aldrich, MO, USA, cat. n. C6628). Chemicals were added to cell cultures at the final concentrations of 10 and 20 μM (4μ8C), 6 and 12 μM (Ceapin) and 10 and 20 ng/mL (GSK2606414) for 24 h; Cloroquine was used at final concentration of 10 μM for 18 h. After treatments, cells were collected, counted by trypan blue exclusion assay using a hemocytometer and used for further analysis. Each experiment was performed in triplicate and repeated at least three times.

### 2.2. Cell Viability

Cell viability was evaluated by a trypan blue (Sigma-Aldrich, MO, USA) exclusion assay after 24 h of culture. Cells were counted by light microscopy using a Neubauer emocytometer. The experiments were performed in triplicate and repeated at least three times.

### 2.3. Western Blot Analysis

After treatments, cells were harvested, centrifuged and finally lysed in modified RIPA buffer (150 mM NaCl, 1% NP40, 50 mM Tris–HCl pH 8, 0.5% deoxycholic acid, 0.1% SDS, 1% Triton X-100 protease and phosphatase inhibitors) on ice for 30 min [14]. Protein concentration was determined by using a BCA protein assay kit (Sigma-Aldrich, MO, USA, 71285-M), and the same amount of each lysate (8–10 μg) was loaded on 4–12% NuPage Bis Tris gels (Thermo Fisher, NP0323). Following electrophoresis, samples were blotted on Nitrocellulose membranes (Whatman, GE Healthcare, 10401196) that were blocked for 30 min at RT in PBS containing BSA 3% and 0.2% Tween 20, then probed with suitable primary antibody overnight at 4 °C. After three washes with PBS 0.2% Tween, membranes were incubated with appropriate secondary HRP-conjugated secondary antibodies (Santa Cruz Biotechnology, Inc., Heidelberg, Germany) for 30 min at RT and finally washed as described above. Membranes were finally subjected to ECL reaction with a blotting substrate (Advansta, CA, USA, 12045-D20).

### 2.4. Antibodies

The following primary antibodies were used for Western blot analysis: rabbit polyclonal anti-PARP1(1:1000) (Proteintech, Rosemont, IL, USA, #13371-1), rabbit polyclonal anti-phospho STAT3 Tyr705 (1:500) (Santa Cruz Biotechnology, Inc. Heidelberg, Germany, #sc-8059), mouse monoclonal anti-STAT3 (1:100) (BD Transduction Lab, Franklin Lakes, NJ, USA, #610189), rabbit polyclonal anti-LC3I/II (1:1000) (Novus, CO, USA, #NB100-2220), mouse monoclonal anti-p62/SQSTM1 (1:300) (BD Transduction Lab, #610832), rabbit polyclonal anti-XBP1 (1:1000) (NovusBio, #NBP1-77681SS), rabbit polyclonal anti-ATF6 (1:200) (Cell Signaling Technology, Danvers, MA, USA, #65880), rabbit polyclonal anti-phospho eIF2α (Ser15) (1:200) (Cell Signaling, #3398), rabbit polyclonal anti-eIF2α (1:500) (Cell Signaling, #9722), mouse monoclonal anti-β-actin (1:10000) (Sigma Aldrich, #A5441) and anti hsp70 (Santa Cruz Biotechnology, Inc. Heidelberg, Germany, #sc-32239) were used as loading control. Goat anti-rabbit IgG-horseradish peroxidase HRP (1:10000) (Santa Cruz Biotechnology, Inc. Heidelberg, Germany, sc-2004), goat anti-mouse IgG-horseradish peroxidase HRP (1:10000) (Santa Cruz Biotechnology, Inc. Heidelberg, Germany, sc-2005) were used as secondary antibodies. All primary and secondary antibodies used in this study were diluted in a PBS-0.2% Tween 20 solution containing 3% BSA.

### 2.5. Densitometric Analysis

Densitometric analysis of Western blot bands was performed by using the ImageJ software, which was downloaded from the NIH web site (http://imagej.nih.gov, version 1.41o, NIH, USA).

### 2.6. Sub-G1 Cell Cycle Analysis

For cell cycle analysis, the DNA content was measured by Propidium Iodide (Sigma Aldrich, USA; P4170) staining and FACS analysis. PEL cells untreated (CT) and treated with GSK (20 ng/mL), 4μ8C (20 μM) and Ceapin (12 μM) for 24 h were washed with cold 1× PBS and fixed in 70% ethanol on ice for at least 1 h. After centrifugation, each cell pellet was washed with cold 1× PBS and stained with 50 μg/mL PI and RNase for 15 min at 37 °C. DNA content was measured by a BD Biosciences FACSCalibur. Data are representative of at least three independent experiments.

### 2.7. Chemiluminescen Immunometric Assay

After 24 h in which BC3 and BCBL1 were cultured with 4μ8C 20 μM or Ceapin 12 μM or GSK2606414 20 ng/mL or untreated (CT), cells were centrifuged, and supernatants were collected to measure Interleukin-10 (IL-10), Interleukin-6 (IL-6) and VEGF by Magnetic Luminex assay performed by R&D systems a Bio-Techne brand, using a human premixed multi-analyte kit (R&D systems Bio-Techne, LXSAHM) according to the manufacturer’s instructions.

### 2.8. RNA Interference

XBP1 silencing was performed by using specific small interfering RNA, according to the manufacturer’s instructions (Santa Cruz Biotechnology, Inc., Heidelberg, Germany, sc-38627). Briefly, 3 × 10^5^ cells were seeded in six-well culture plates in RPMI medium without antibiotics for 24 h. Next, 100 pmol of specific small interfering RNA (siRNA duplex) or non-targeting (scramble) siRNA and 10 μL of transfection reagent (Lipofectamine 2000; Invitrogen, CA, USA, 11668-027) were diluted in Opti-MEM medium (Thermo Fisher, Waltham, MA, USA, 31985062) and added to the cells according to the manufacturer’s instruction [15]. After 24 h, cells were collected, and the viability was assessed by trypan blue exclusion assay. Cells were harvested and used for Western blot analysis. Each experiment was performed in triplicate.

### 2.9. Statistical Analysis

Results are represented by the mean ± standard deviation (SD) of at least three independent experiments, and a two-tailed Student’s *t*-test was used to demonstrate statistical significance. A difference was considered as statistically significant when the *p*-value was at least <0.05 (*).

## 3. Results

### 3.1. The Inhibition of IRE1alpha/XBP1 Axis by 4μ8C Strongly Reduces PEL Cell Survival While GSK2606414 PERK or Ceapin ATF6 Inhibitors Slightly Affect it

**Figure 1:**
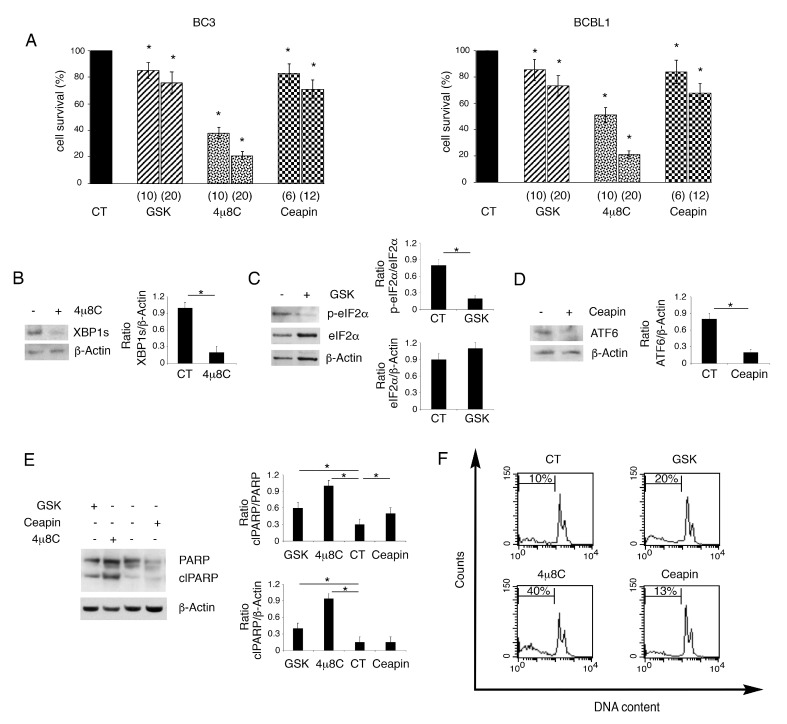
4μ8C strongly impairs PEL cell survival while GSK2606414 or Ceapin slightly reduce it. BC3 and BCBL1 were cultured for 24 h with GSK2606414 (GSK), 4μ8C and Ceapin at two concentrations or without (CT), and (**A**) cell survival was evaluated by trypan blue exclusion assay. The histograms represent the percentage of cell viability relative to the control; data are represented as the mean plus SD of more than 3 experiments * *p* value<0.05. The expression of XBP1 (**B**), p-eIF2alpha and eIF2alpha (**C**) and ATF6 (**D**) inhibited by their respective inhibitor 4μ8C (20 μM), GSK (20 ng/mL) and Ceapin (12 μM) was evaluated by Western blot analysis in BCBL1 PEL cells. Actin was used as loading control. A representative experiment out of three is shown. Histograms represent the mean plus SD of the densitometric analysis of the ratio of specific protein/β-actin. (**E**) Cleavage of PARP was evaluated by Western blot analysis after treatment of PEL cells with GSK (20 ng/mL), 4μ8C (20 μM) and Ceapin (12μM) or without (CT). Actin was used as loading control. A representative experiment out of three is shown. Histograms represent the mean plus SD of the densitometric analysis of the ratio of clPARP/PARP and clPARP/β-actin. * *p*-value < 0.05. (**F**) sub-G1of untreated (CT) and treated with GSK (20 ng/mL), 4μ8C (20 μM) and Ceapin (12 μM) PEL cells was evaluated by FACS analysis after staining with PI. % of sub G1 is reported inside the histograms.

Previous studies have shown that PEL cells display a basal UPR activation [6], as they are secretory B cells characterized by a high level of ER stress. Therefore, we first inhibited IRE1alpha endoribonuclease activity by using 4μ8C. We found that it induced a dose-dependent cell death in both BC3 and BCBL1 PEL cells (Figure 1A). Differently from 4u8c, the inhibition of PERK by GSK2606414 or of ATF6 by Ceapin slightly reduced PEL cell survival (Figure 1A). We then evaluated whether these inhibitors at the doses used to treat PEL cells could effectively reduce the activation of the three UPR sensors. Therefore, we evaluated by Western blot analysis the spliced form of XBP1 (XBP1s), target of IRE1alpha endoribonuclease activity, the phosphorylation of eIF2alpha, target of PERK and the cleaved form ATF6, whose formation is inhibited by Ceapin, as it antagonizes ATF6 transport to the Golgi apparatus [16]. As shown in Figure 1B–D, the expression level of XBP1s and the cleaved form of ATF6 as well as the phosphorylation of eIF2alpha were reduced by using the UPR specific inhibitors, used at the highest dose. To explore whether the impairment of cell survival occurring following UPR inhibition, particularly following 4μ8C treatment, could be due to apoptosis induction, we evaluated the cleavage of PARP1, as it can be mediated by caspase activation [17]. As shown in Figure 1E, the PARP1 cleaved form increased in PEL cells treated by 4μ8C, while it slightly increased following treatment with GSK2606414 or Ceapin. Apoptosis induction was confirmed by the increase in subG1 events that was particularly evident in 4μ8C-treated PEL cells (Figure 1F).

### 3.2. 4μ8C Reduces the Release of Pro-Inflammatory/Immune Suppressive Cytokines and STAT3 Activation in PEL Cells

**Figure 2:**
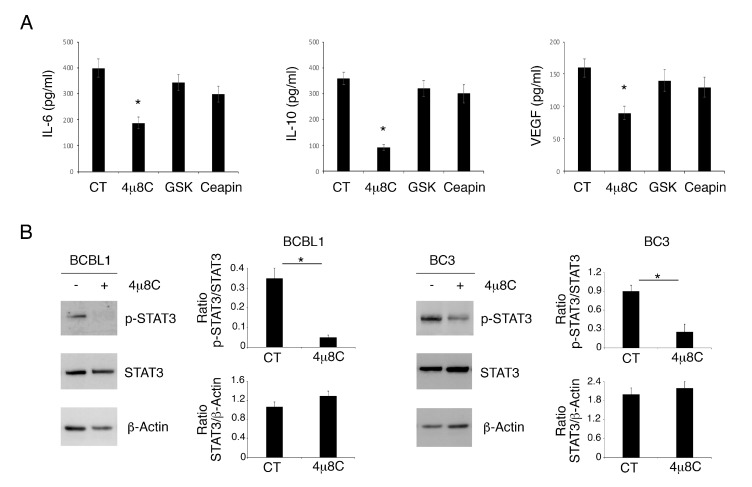
4μ8C reduces pro-inflammatory/immune suppressive cytokines and inhibits STAT3 phosphorylation in PEL cells. Release of IL-6, IL-10 and VEGF was measured in supernatants of PEL cells treated with 4μ8C (20 μM), GSK (20 ng/mL) and Ceapin (12 μM) or without (CT), (**A**). Histograms representing the mean plus SD of the amount of IL-6, IL-10 and VEGF of three independent experiments are shown. * *p*-value < 0.05. (**B**) The expression of p-STAT3 and STAT3 in BCBL1 and BC3 cells treated or not (CT) with 4μ8C (20 μM) was evaluated by Western blot analysis. Actin was used as loading control. A representative experiment out of three is shown. Histograms represent the mean plus SD of the densitometric analysis of the ratio of p-STAT3/STAT3 and STAT3/β-actin. * *p*-value < 0.05.

Searching for the molecular mechanism/s that could lead to higher cell death induction by 4μ8C, we assessed the release of cytokines IL-6, IL-10 and VEGF, known to sustain PEL cell survival following treatment with 4μ8C or GSK2606414 or Ceapin. The results shown in Figure 2A indicate that only 4μ8C strongly reduced the production of these cytokines, which was slightly downregulated by GSK2606414 or Ceapin. These cytokines may lead to the activation of STAT3, a pathway whose constitutive phosphorylation plays a pro-survival role in PEL. We found that STAT3 705 tyrosine phosphorylation (*Tyr705)* was reduced by 4μ8C (Figure 2B), according to its mediated impairment of cytokine release. All together, these results suggest that the reduction in cytokine production and STAT3 de-phosphorylation could underlie the impairment of PEL cell survival mediated by 4μ8C.

### 3.3. 4μ8C Induces Autophagy Whose Inhibition Increases Its Cytotoxicity against PEL Cells

**Figure 3:**
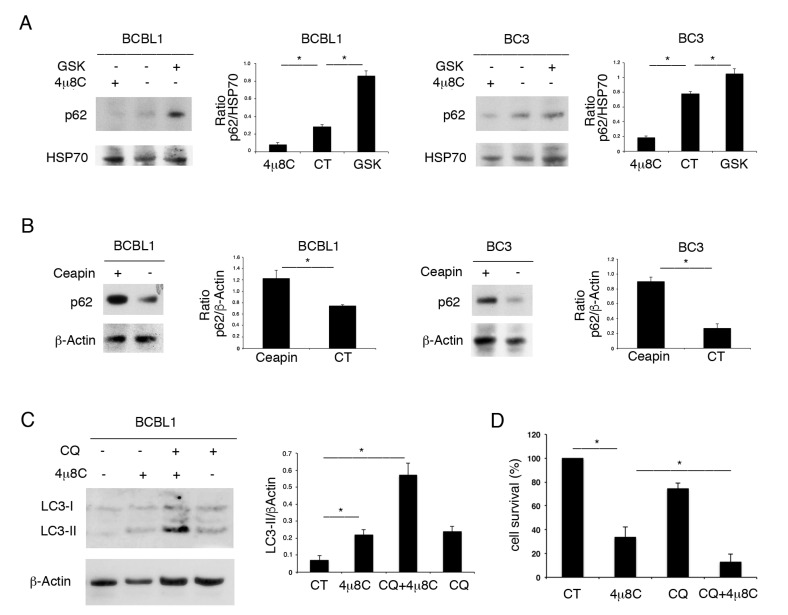
4μ8C promotes a pro-survival autophagy in PEL cells. (**A**) BC3 and BCBL1 cells were treated with 4μ8C or with GSK2606414 (GSK) or (**B**) with Ceapin and p62/SQSTM1 (p62) expression level was evaluated by Western blot. (**C**) LC3I/II was evaluated by Western blot analysis in BCBL1 PEL cells treated by 4μ8C in the presence or in the absence of the autophagy inhibitor Cloroquine (CQ). A representative experiment is shown. Histograms represent the mean plus SD of the densitometric analysis of three independent experiments. * *p*-value < 0.05. (**D**) Cell survival was evaluated by trypan blue exclusion assay in PEL cells treated by 4μ8C in the presence or in the absence of the autophagy inhibitor Cloroquine. Histograms represent the mean plus SD of three independent experiments. * *p*-value < 0.05.

We then evaluated the impact of UPR sensor inhibitors on autophagy. As shown in Figure 3A,B, while 4μ8C reduced the expression level of p62/SQSTM1, GSK2606414 and Ceapin promoted its accumulation. As p62/SQSTM1 is mainly degraded through autophagy, these results suggest that 4μ8C promoted the autophagic flux, while the other two UPR inhibitors reduced it. The induction of autophagy by 4μ8C was confirmed by its combination with Cloroquine, a drug that inhibits the late autophagic steps. We found that the expression level of the lipidated form of LC3 (LC3II) increased with 4μ8C and further accumulated by using Cloroquine (Figure 3C). As LC3II is formed and degraded through autophagy, preventing its degradation by Cloroquine allows us to evaluate LC3II formation [18]. Therefore, the higher LC3II increase in the presence of 4μ8C plus Cloroquine, together with the reduction in p62 expression level, suggests that 4μ8C induced a complete autophagic flux in PEL cells. We took advantage of using Cloroquine to also evaluate the role of autophagy activation by 4μ8C on cell survival. As shown in Figure 3D, PEL cell survival impaired by 4μ8C was further reduced by its combination with Cloroquine, suggesting that autophagy activation by 4μ8C represented a pro-survival mechanism.

### 3.4. XBP1 Silencing Induces Autophagy and Reduces Cells Survival in PEL Cells

**Figure 4:**
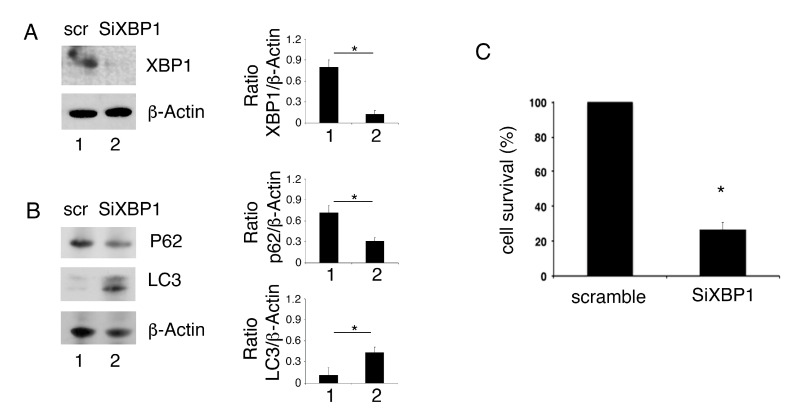
XBP1 knock-down promotes autophagy and impairs cell survival in PEL cells. The expression of XBP1, p62 and LC3 was evaluated by Western blot analysis in PEL cells silenced with siRNA XBP1 and control cells (scramble, scr) (**A**,**B**). Actin was used as loading control. A representative experiment out of three is shown. Histograms represent the mean plus SD of the densitometric analysis of the ratio of XBP1/β-actin, p62/β-actin and LC3/β-actin. * *p* value<0.05. (**C**) cell survival of PEL cells silenced with siRNA XBP1 and control cells (scramble) was evaluated by trypan blue exclusion assay. The histograms represent the percentage of cell viability relative to the control; data are represented as the mean plus SD of more than 3 experiments * *p* value<0.05.

The role of XBP1 inhibition in autophagy and survival of PEL cells was then evaluated by silencing it by specific siRNA. We found that XBP1 knock-down (Figure 4A) reduced p62/SQSTM1 expression level in comparison with scramble-treatment, suggesting autophagy activation by XBP1 silencing (Figure 4B). Moreover, similarly to what was observed by using 4μ8C, XBP1 silencing increased LC3II expression level (Figure 4B) and reduced PEL cell survival (Figure 4C).

## 4. Discussion

Tumor cells, especially those that are highly secretory, such as the cancer B cells belonging to Multiple Myeloma (MM) or PEL, are characterized by a high level of basal ER stress and a constitutive UPR activation. As such, these cells are very sensitive to ER perturbation; therefore, they may be successfully treated with drugs that exacerbate ER stress, such as HSPs inhibitors [19] that reduce the folding capacity of the cells or Bortezomib [7], which, by inhibiting the proteasome, further increases the already high protein load in the ER. However, UPR may represent a rational drug target for cancer that strongly relies on its activation, and indeed UPR inhibition may be a promising anticancer strategy [20,21], considering that UPR triggers pro-survival processes such as the upregulation of chaperones or autophagy. However, drugs that exacerbate ER stress/UPR can also have a cytotoxic effect on cancer cells [22], suggesting that the activation of UPR in stressed cancer cells must be well balanced as it may drive cells towards either death or survival, depending on the duration, intensity and nature of stress. Of note, the response initiated by the three UPR sensors is interconnected either in a positive way, as when they converge on the activation of the same targets, or in a negative way, as the activation of a sensor may inhibit the other/s [23,24].

In this study, we found that PEL cells were more highly dependent on the function of the IRE1alpha/XBP1 axis rather than PERK and ATF6 branches. Indeed, IRE1alpha/XBP1 axis inhibition by 4μ8C decreased the production of cytokines such as IL-6, IL-10 and VEGF, while such process was slightly influenced by GSK2606414 or Ceapin, PERK and ATF6 inhibitors. The dependence of PEL from the release of these cytokines for its survival has been clearly shown in previous studies [25]. Indeed, it must be considered that these cytokines may activate, in an autocrine fashion, transcription factors such as STAT3 in PEL cell [26] and that its constitutive activation plays a key role in sustaining PEL survival [4]. Accordingly, here, we found that in addition to impairing cytokine production, Ire1alpha/XBP1 axis inhibition led to a reduction in STAT3 phosphorylation in these cells. The close relationship between IRE1alpha/XBP1 axis activation and pro-inflammatory/immune suppressive cytokine secretion has been recently shown by our laboratory in macrophages infected by KSHV [27], the same virus carried by PEL cells and involved in the etiology of this lymphoma. Moreover, IL-6, IL-10 and VEGF, produced by cancer cells, may activate STAT3 in a paracrine fashion in the cells present in the tumor microenvironment, such as myeloid cells or fibroblasts, transforming them into cells that support tumor growth [28]. Therefore, it will be important, in future studies, to evaluate the consequence of IRE1alpha/XBP1 axis inhibition on the cross-talk between PEL cells and cells of the tumor environment or whether IRE-XBP1 signaling inhibition may interfere with HIF1α. Indeed, it is known that the interaction between these pathways may sustain cancer growth in hypoxic condition or promote the epithelial to mesenchymal transition (EMT) [29]. However, the importance of targeting the IRE1alpha/XBP1 axis in vivo and in vitro has been reported by a variety of studies, particularly those focused on hematological cancers. In addition to 4μ8C, several small molecules inhibiting this axis, such as MKC-3946 and STF-083010, have been used [30,31]. Another important finding of this study was that IRE1alpha/XBP1 axis inhibition triggered autophagy in PEL cells and that its inhibition by Cloroquine could further promote PEL cell death. Inhibiting autophagy, particularly in the course of anticancer treatments, such as those that inhibit STAT3, has been previously shown to promote PEL cell death [4,5]. Indeed, it known that autophagy may help cells to adapt to stressful conditions including anticancer treatments. Based on this evidence, various drugs targeting both UPR and autophagy are currently in preclinical and clinical trials [32]. As autophagy also influences the release of Damage Associated Molecular Patterns (DAMPs) [33], it will be also important to evaluate how the IRE-XBP1 axis may shape the tumor environment by modulating autophagy [34,35].

In conclusion, this study suggests that targeting UPR, particularly the IRE1alpha/XBP1 axis, may represent a promising strategy against PEL and that its cytotoxic effect may be increased by combing it with autophagy inhibitors.

## Data Availability

The data presented in this study are available on request from the corresponding author.

## Abbreviations

Activating transcription factor (ATP); Endoplasmic Reticulum (ER); Epstein-Barr virus (EBV); Fas-associated with death domain (FADD)–like interleukin-1β–converting enzyme (FLICE)/caspase 8–inhibitory protein (FLIP); interleukin (IL); inositol requiring kinase (IRE); Kaposi’s Sarcoma-associated Herpesvirus (KSHV); Microtubule-associated protein 1A/1B-light chain 3 (LC3); multiple myeloma (MM); Nuclear factor-κB (NF-kB); nuclear factor erythroid 2-related factor (NRF2); p62, sequestosome 1 (SQSTM1); Poly (ADP-ribose) polymerase 1 (PARP1); Primary Effusion Lymphoma (PEL); double stranded RNA-activated protein kinase-like ER kinase (PERK); Signal Transducer and activator of Transcription 3 (STAT3); Unfolded Protein Response (UPR); Vascular-Endothelial Growth Factor (VEGF); X-box binding protein 1 (XBP1).
